# Investigation of antimicrobial activity of photothermal therapeutic gold/copper sulfide core/shell nanoparticles to bacterial spores and cells

**DOI:** 10.1186/1754-1611-8-11

**Published:** 2014-06-02

**Authors:** Ebenezer Addae, Xiuli Dong, Eric McCoy, Chang Yang, Wei Chen, Liju Yang

**Affiliations:** 1 Department of Pharmaceutical Sciences, Biomanufacturing Research Institute and Technology Enterprises (BRITE), North Carolina Central University, Durham, NC 27707, USA; 2 Department of Physics, University of Texas at Arlington, Arlington, TX 76019, USA; 3 Guizhou Provincial Key Laboratory of Pharmaceutics, School of Pharmacy, Guiyang Medical College, Guiyang, Guizhou 550004, China

**Keywords:** *Bacillus anthracis*, Nanoparticles, Bioterrorism, Antimicrobial

## Abstract

**Background:**

Au/CuS core/shell nanoparticles (NPs) were designed as a new type of transducer agent for photothermal therapy (PTT), with attractive features of easy preparation, low cost and small size for targeting. This paper studied for the first time the intrinsic antimicrobial activity of Au/CuS NPs to *B. anthracis* spores and cells in addition to its PTT effect.

**Results:**

It was found that Au/CuS NPs were highly efficient in inactivating *B. anthracis* cells, but not effective to the spores. Treatment with NPs at ~0.83 μM for 30 min achieved a 7 log reduction in viable cells. The antimicrobial effect was both NPs concentration and treatment time dependent. SEM imaging and the efflux of DNA test demonstrated the damage of cell membrane after NPs treatment, yet further research is necessary to fully understand the precise inactivation mechanism.

**Conclusions:**

The Au/CuS NPs had strong antimicrobial activity to *B. anthracis* cells, which showed a great potential to be an effective antimicrobial agent to bacterial cells.

## Background

Various nanoparticles (NPs) are a class of nanomaterials that has emerged in the nanotechnology era. With their distinct physical and chemical properties, NPs have gained increasing attention in numerous applications in the biological, biomedical, physical and pharmaceutical fields. Studies have shown that antimicrobial formulations made from a variety of NPs have high bacteriocidal activity [1,2]. For example, Klabunde and co-workers recently reported that dry powder formulations and slurries of magnesium oxide NPs have great biocidal effect on both gram positive and gram negative bacteria and their spores [3]. Sondi and Salopek-Sondi reported that silver NPs kill *E. coli* cells by penetrating the cell membrane [2]. Zawrah and El-Moez discovered that Au NPs exhibited strong antimicrobial activity to various foodborne pathogens. They also found that drugs coated with NPs were highly effective against *C. albicans, A. niger and A. flavus* and that the coating of Au NPs minimized treatment durations and side effects of the drugs [4]. The mechanism of how metal NPs inactivate bacterial cells involves either destruction of the cell wall or membrane integrity [5-7]. The negatively charged bacterial cells are believed to cultivate electrostatic interactions between the cells and the positively charged NPs resulting in the compromise of the cell membranes, and eventually cell death [8].

Au/CuS core/shell NPs were initially designed as a new type of transducer agent for photothermal therapy (PTT) of cancer because the core/shell structure combines the advantageous features of CuS – easy preparation, low cost and small size for targeting – with enhanced PTT efficiency through Au NP surface plasmon. Sun et al. first reported the synthesis of Au/CuS core/shell NPs [9], and Lakshamanan et al. first demonstrated their PTT efficacy in cancer treatment [10]. Aside from their PTT effect, based on the potential of metal and other NPs as bactericides, we postulate the possibility of using Au/CuS NPs as a bactericidal agent against bacterial spores and cells.

*Bacillus anthracis* is a gram positive, rod shaped, non-motile and spore forming bacterium. It causes anthrax - a life-threatening disease primarily found in herbivores, but it also affects other mammals, including humans [11-13]. *B. anthracis* enters into hosts through three main routes, namely cutaneous (through skin abrasions or skin lesions caused by biting insects), gastrointestinal (by the ingestion of spore contaminated food, water or forage) or pulmonary (by the inhalation of dust that contain spores). Upon entry, *B. anthracis* spores travel to lymph nodes where they germinate into vegetative bacilli that produce the characteristic virulence factors – the toxin and the capsule (encoded for by two plasmids pX01 and pX02, respectively) – and enter into blood circulation. Once in the blood, the vegetative cells multiply rapidly and continue to produce the toxin until it eventually subdues the host system and causes a shock-like death. When bacilli from the dying or dead host are exposed to the air (oxygen), they sporulate and the cycle begins all over again [11-13]. In the 2001 bioterrorism attacks in the United States of America, terrorists mailed letters containing dry *B. anthracis* spores to people in the news media and government, which ultimately led to five deaths, about 30,000 people being treated with antibiotics, and numerous public buildings being decontaminated [14]. According to the FBI (Federal Bureau of Investigation), the attack cost over one billion dollars in damages with cleanup costs contributing about three hundred and twenty million dollars [15]. After the 2001 attack, federal agencies used chlorine dioxide gas, vaporized hydrogen peroxide, para-formaldehyde and gamma radiation to decontaminate the buildings affected [15,16], but these antimicrobial agents are not effective and some of them may themselves pose harm to first responders. For instance, formaldehyde is known to play a possible role in carcinogenesis [16]. The 2001 attack has heightened the attention of researchers to find efficient, cost effective ways to decontaminate environments inhabited by *B. anthracis* spores and cells. Whitney et al. summarized all the methods for inactivating *B. anthracis* spores and cells from available literature, and pointed out that there is insufficient scientific knowledge on decontaminating buildings after the intentional release of *B. anthracis* spores [16]. Therefore, there is a need to explore some of the newly discovered technologies/materials for applications in such circumstances against anthrax infections and spore contamination [15,16].

In this study, we investigated the effectiveness of Au/CuS NPs for inactivating *B. anthracis* spores and cells considering variables such as Au/CuS concentration, treatment medium, and treatment time. We also examined the interactions between Au/CuS NPs and *B. anthracis* cells and spores using fluorescence microscopy, scanning electron microscopy (SEM) and energy dispersive X-ray spectroscopy (EDS). The possible antimicrobial mechanism of Au/CuS on *B. anthracis* cells and spores was also discussed.

## Results and discussion

### Effect of Au/CuS NPs pre-treatment and continuous presence on the growth of *B. anthracis* spores

**Figure 1:**
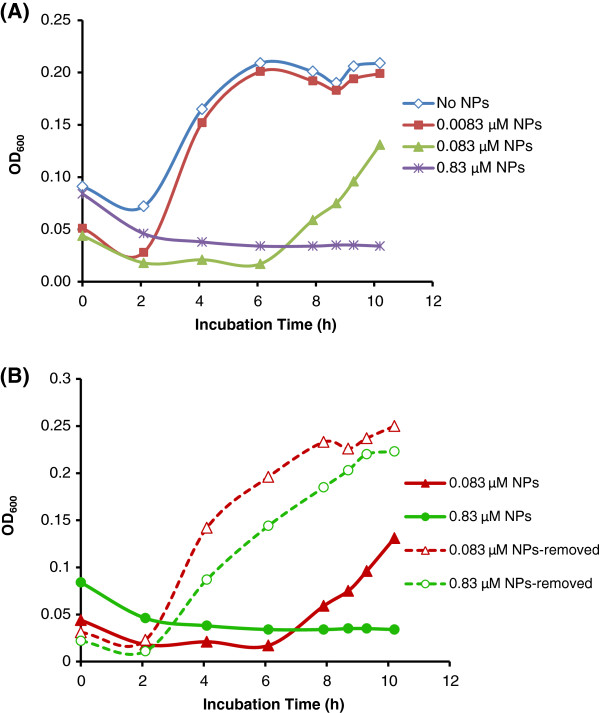
**The effects of Au/CuS NPs’ pre-treatment and continuous presence in medium on the growth of *B. anthracis *spores. (A)** The representative growth curves of *B. anthracis* spores after being pre-treated with 0.0083 μM, 0.083 μM and 0.83 μM Au/CuS for 30 min and subsequent growth in nutrient broth without removing the NPs. **(B)** The comparison of the growth curves of *B. anthracis* spores pre-treated with 0.083 μM, 0.83 μM AuCuS NPs, when the NPs were removed before growth and when the NPs were in the presence in the growth medium. Initial spore concentration: ~10^7^ spores/mL, pre-treated with NPs in DI water for 30 min.

The growth of *B. anthracis* spores treated with 0.0083 μM to 4.15 μM Au/CuS NPs were monitored by optical density (OD) measurement. Figure 1A shows the representative growth curves of *B. anthracis* spores (~10^7^ spores/mL) after being pre-treated with 0.0083, 0.083 and 0.83 μM Au/CuS for 30 min and subsequent growth in nutrient broth without removing the NPs. The control was the spore sample without NPs treatment. As shown in Figure 1A, the control started the exponential growth phase after approximately 2.1 h of lag phase. The spore sample pre-treated with 0.0083 μM of NPs showed growth similar to the control, while the spore sample pre-treated with 0.083 μM of NPs started its exponential phase at approximately 6.1 h, much later than the control, and the sample pre-treated with 0.83 μM of NPs did not show growth up to 10 h incubation. In repeated tests, every sample showed this growth pattern. The difference in the initial absorbance and the absorbance in the early stage of growth between the samples was contributed by the increased concentration of NPs (yellowish colored). However, this difference did not affect the overall shape of the growth curve which clearly included the lag phase, the exponential phase, and the stationary phase. When the growth curves of these samples were compared, it was observed that the durations of the lag phase were different. Prolonged lag phases could be due to the reduced viable spore number in each NPs treated sample [17,18]. It was also possible that the presence of NPs in the medium inhibited the spore growth at any stage during the growth. The results suggested that either the reduction of viable spores by the pre-treatment with NPs or the presence of NPs in the growth medium, or both, inhibited the growth of spores. It also indicated that the extent of inhibition was related to the concentration of NPs in the medium.To further investigate which process was the cause of growth inhibition, we did growth tests on the pre-treated spore sample after removal of NPs. Figure 1B shows the comparison between the growth curves of NPs pre-treated spores with and without the removal of NPs in the growth medium. It is clear that without the removal of NPs, for the spores pre-treated with 0.083 and 0.83 μMNPs for 30 min, the growth were partially (0.083 μM) or fully (0.83 μM) inhibited within the 10 h incubation period. However, when the NPs were removed after 30 min pre-treatment and before the spore growth in the medium, the growth curve returned to a pattern similar to that of the untreated control sample (shown in Figure 1B). This observation indicated that the inhibition of spore growth was primarily caused by the presence of Au/CuS NPs in the growth medium rather than the pre-treatment with NPs. It should also be noted that the actual concentrations of the NPs presented in the growth medium were one order lower than those in the pre-treatment solutions, i.e. the presence of 0.0083 μM of NPs in the growth medium caused a significantly prolonged lag phase (from ~2.1 h to ~6.1 h), and 0.083 μM of NPs in the growth medium fully inhibited the spore growth during the 10 h testing period (Figure 1A).

### Pre-treatment with Au/CuS NPs did not effectively inactivate *B. anthracis* spores

**Figure 2:**
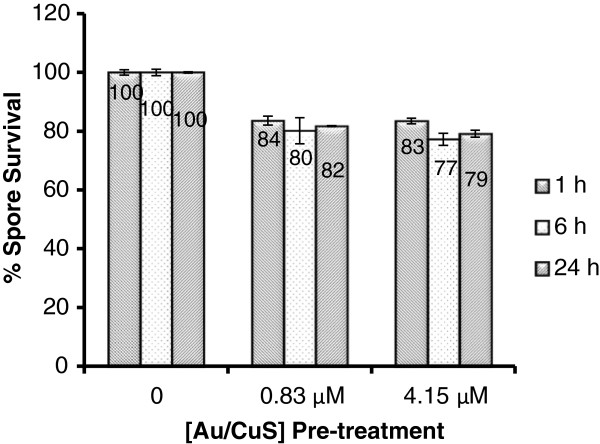
**The percentages of surviving *B. anthracis *spores upon pre-treatment with Au/CuS NPs at 0.83 μM and 4.15 μM and treatment durations varying from 1 h to 24 h.** Initial spore concentration: 10^5^ spores/mL in DI water.

To further look into the effect of NPs pre-treatment, we examined the reduction of viable spores in the NPs pre-treated samples using the traditional plating method. Figure 2 shows the percentage of survival spores after pre-treatment with 0.83 and 4.15 μM of Au/CuS NPs for 1, 6, and 24 h. Pre-treatment with 0.83 μM Au/CuS NPs to *B. anthracis* spores in DI water suspension for 1 h only inactivated about 16% of the spores in the sample containing 3.98 × 10^5^ spores/mL, whereas in the growth test, the presence of 1/10 dilution of this concentration of NPs in growth medium inhibited the spore growth during the 10 h incubation period. With the same NPs concentration (0.83 μM), even if the pre-treatment time prolonged to 6 h and 24 h, still only 18-20% spores were inactivated during pre-treatment. With the same treatment time (1 h), a higher concentration of NPs (4.15 μM) in the pre-treatment caused ~17% of spores, which was similar to that of the 0.83 μM NPs pre-treatment. Similarly, at this higher concentration of NPs, prolonged pre-treatment time to 6 h and 24 h did not significantly increase the inactivation efficiency (17-23% inactivation). The results indicated that Au/CuS NPs pre-treatment was not effective in inactivating *B. anthracis* spores, and neither the increasing NPs treatment time nor the increasing concentration of NPs produced substantial spore inactivation effect.

**Figure 3:**
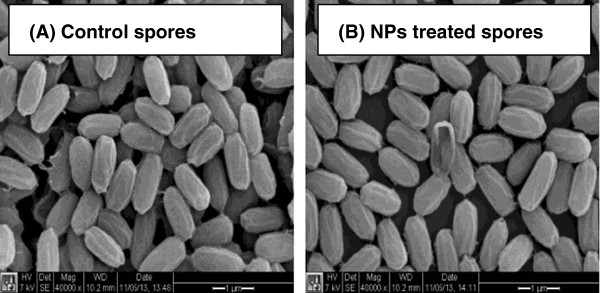
**SEM images of untreated *B. anthrcis *** **spores (A) and spores treated with 4.15 μM Au/CuS NPs for 1 h (B).**

SEM imaging was used to further examine the morphology of the NPs-treated spores. Figure 3 shows the SEM images of Au/CuS NPs treated and untreated spores. No significant difference in morphology was observed between the NPs-treated and the untreated spores. EDS analysis showed the presence of Cu and S elements on the NPs treated spores sample which indicated that the Au/CuS NPs did have contact with the spores and remained on the surface of spores, whereas the untreated spores did not show the peak of Cu element in the sample (Additional file 1: Figure S1). It was not surprising that element S presented in both untreated and NPs treated samples as biological samples usually contain element S in their components.

The SEM and EDS results suggested that Au/CuS NPs did interact with *B. anthracis* spores during pre-treatment, but this pre-treatment was not effective in inactivating spores. Considering the structure of a spore, the resistance of spores to Au/CuS NPs treatment is understandable. The spore is usually encased in a thick multilayered coat surrounded by the exosporium [19], making the spore the most resistant life form known. In fact, research has demonstrated that *B. anthracis* spores are much more resistant than their vegetative cell counterparts to a variety of treatments [20].

However, despite the spores’ resistance to NPs treatment, the results showed promise that the presence of Au/CuS NPs in the growth medium was effective in inhibiting the outgrowth of spores at one or more of the stages from spore to vegetative cell. There are a series of steps happening during the process of turning dormant spores into growing vegetative cells, typical including germination stage I & II, and outgrowth stage. The steps include the release of H^+^, monovalent cations and Zn^2+^, the release of dipicolinic acid and its associated Ca^2+^, the core hydration, the hydrolysis of spore cortex, further swelling of the spore due to further water uptake and the expansion of the germ cell wall. After germination stage I and II, the enzyme activity, metabolism activity, macromolecule synthesis activity are activated, spores enter to outgrowth stage in which they shed their exosporium and return to the vegetative cell form [21]. Most likely, the NPs functioned to inactivate the vegetative cells that were outgrown from the spores. In this context, we further investigated the antimicrobial effect of Au/CuS NPs on *B. anthracis* cells.

### Au/CuS NPs treatment effectively inactivated *B. anthracis* cells

**Figure 4:**
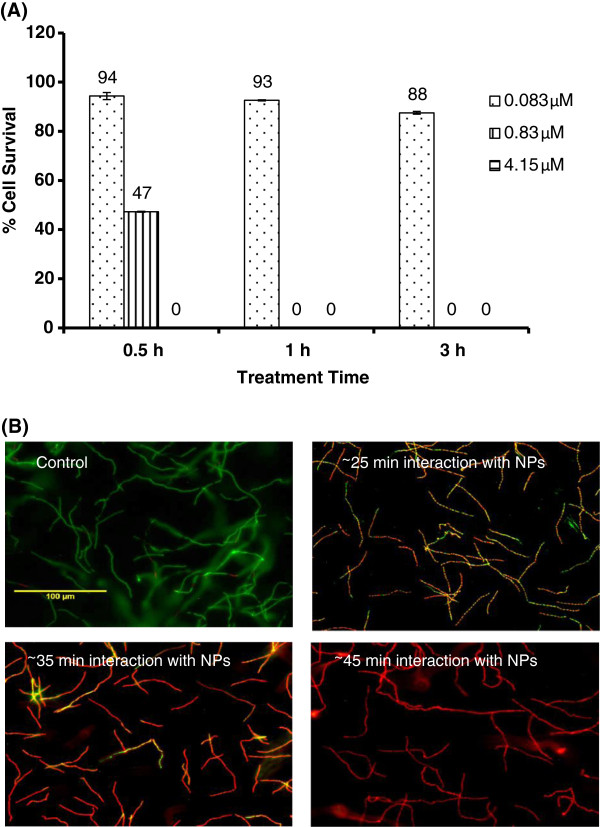
**The effect of Au/CuS NPs pre-treatment on *B. anthracis *cells. (A)** The percentages of surviving *B. anthracis* cells upon the treatment with Au/CuS NPs at 0.083 μM, 0.83 μM, and 4.15 μM and treatment duration varying from 0.5 h to 3 h. Initial cell concentration: 1.6 ×10^7^ cells/mL in DI water. **(B)** The fluorescence images of the control *B. anthracis* cells and cells interacted with Au/CuS NPs in DI water for ~25 min, ~35 min and ~45 min. Cells were stained with the Live/dead BacLight bacteria staining kit, live cell in green, and dead cells in red.

The traditional plating method was used to determine the viable cell number in *B. anthracis* cell samples treated with various concentrations of Au/CuS NPs for different treatment times. Figure 4A shows the survival cell percentage after the treatment with 0.083, 0.83 and 4.15 μMAu/CuS NPs for 30 min to 3 h. NPs concentration as low as 0.083 μM was not very effective in inactivating *B. anthracis* cells, as 30 min to 3 h treatment with 0.083 μM of NPs only inactivated 6% to 12% of the cells in the samples containing 1.6 × 10^7^ cells/mL. Increasing NPs concentration effectively increased the efficiency of NPs treatment in inactivating cells, as treatment with 0.83 and 4.15 μM Au/CuS produced 53% and 100% cell inactivation, respectively, for the same 30 min treatment time.

At a given NPs concentration, the antimicrobial activity of NPs to *B. anthracis* cells showed a clear treatment time dependency. This was well presented by the treatment with 0.83 μM NPs, which produced 53% to 100% cell inactivation when treatment time increased from 30 min to 1 h. The time-dependent inactivation of bacterial cells by Au/CuS NPs was also confirmed by the fluorescence images of cells stained with Live/dead BacLight bacteria staining kit during the course of treatment (Figure 4B). The staining kit contains two nucleic acid dyes—green fluorescent SYTO 9 dye and red-fluorescent propidium iodide (PI) dye. These two components differ in their ability to penetrate healthy bacterial cells. When used alone, SYTO 9 dye stains both live and dead bacterial cells. When both dyes are used, PI penetrates only bacterial cells with damaged membranes, reducing SYTO 9 fluorescence. Therefore, live cells with intact membranes will be stained green, while dead cells with damaged membranes will be stained red [22]. The images in Figure 4B indicated the increase of dead cells with increasing NPs treatment time. *B. anthracis* cells dispersed well in deionized (DI) water suspension (the control) presented as chain-forming cells with almost all live cells (green) and only 1–2 dead cells (red). When the cell suspension was treated with 0.83 μM Au/CuS NPs for 10 min with additional ~15 min processing time (a total of ~25 min), the image showed about 50% cells were dead (red), although some of them appeared as yellow (combination of green and red) since SYTO 9 still stained dead cells. With 20 min treatment time and additional ~15 min processing time (a total of ~35 min), significantly increased dead cells were presented in the sample. With 30 min treatment time and ~15 min processing time (a total of ~45 min), all the cells appeared dead (red). The fluorescence imaging results confirmed the treatment time-dependency of NPs in inactivating bacterial cells. The penetration of PI dye (red) to the cells also indicated the damage to cell membranes after NPs treatment.

The results above demonstrated that the antimicrobial activity of NPs to bacterial cells were NPs concentration and treatment time dependent. With NPs concentrations at 0.83 μM and higher, combination of appropriate NPs concentration and reasonable treatment time (30 min to 1 h) can reach 100% bacterial cell inactivation, i.e. a 7 log reduction in viable cells.

Such high reductions in viable cells demonstrated that the antimicrobial efficiency of Au/CuS NPs is more effective than a variety of other physical and chemical methods and other nanomaterials-based methods reported in literature for inactivation of bacterial pathogens, by which the log reduction of viable cell number ranged from less than 1 to several logs [23-26] and the effectiveness is similar to that of the well known silver NPs, which have been reported that 10 mg/mL Ag NPs completely inhibited the growth of 10^7^ CFU/mL *E. coli* cells in growth medium [27]. Nanomaterials such as single walled carbon nanotubes (SWCNTs) have been reported as a strong antimicrobial agent, but their antimicrobial activity appeared less effective than the Au/CuS NPs. For example, an average of ~80% and ~87% of *E. coli* cells (less than one log reduction) were inactivated by incubating cells with SWCNTs for 60 min and incubating cells on SWCNT-coated filter for 1 h, respectively [28,29]. Since *B. anthracis* infection includes multiple steps, including spore entry and germination, bacillar multiplication and dissemination, and toxin production, destroying spores, cells, and/or toxins in any of these steps is a useful strategy to preventing infection. In addition, the highly effective antimicrobial activity of Au/CuS NPs to *B. anthracis* cells renders the possibility for inactivation of other bacterial pathogens in different applications. A systematic comparison between the performance of the Au/CuS and other metal NPs will be useful for more detailed evaluation and further advancement of its applications in the antimicrobial field.

**Figure 5:**
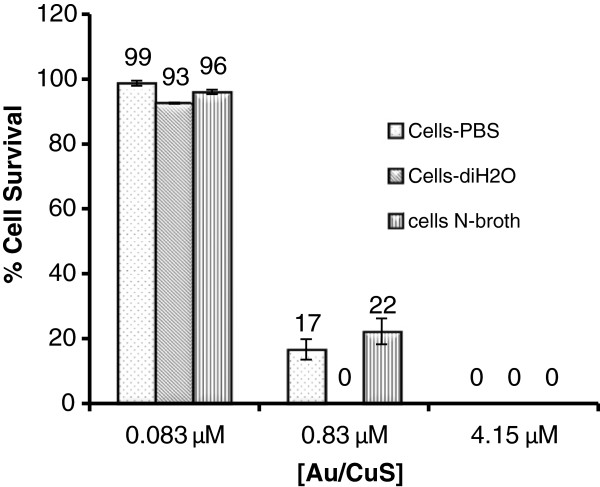
**The comparison of the percentages of surviving cells after *B. anthracis *cells were treated with 0.083 μM, 0.83 μM, and 4.15 μM Au/CuS NPs for 1 h in PBS, DI-H**_**2**_**O and nutrient broth.** Initial cell concentration: (0.79-3.8) ×10^7^ cells/mL.

We also examined the effect of buffer on the antimicrobial activity of Au/CuS NPs to *B. anthracis* cells. In addition to the cell suspension in DI water, we also tested the cell suspensions in 0.1 M PBS and in fresh nutrient broth. Figure 5 compares the percentages of surviving cells after *B. anthracis* cells were treated with 0.083, 0.83 and 4.15 μM Au/CuS NPs for 1 h in PBS, DI-H_2_O and nutrient broth. Treatment with 0.083 μM Au/CuS was ineffective in inactivating *B. anthracis* cells in all three buffers, resulting in only 3–6% cell inactivation. The effectiveness of treatment with 0.83 μM Au/CuS appeared to have a slight association with the buffer in which the treatment was performed, the inactivation percentage was 83%, and 78% in 0.1 M PBS and nutrient broth, respectively, whereas it was 100% cell inactivation in DI water. At higher NPs concentration (4.15 μM), the treatment inactivated 100% of the cells in all three buffers. The results pointed to the fact that the inactivation of *B. anthracis* cells was primarily dependent on the NPs concentration, and the treatment media slightly affected the inactivation efficiency at moderate NPs concentrations. However, the toxicity of Au/CuS NPs to other types of cells, or to human body, and environmental fate of the NPs must be investigated before any practical applications can be advanced.

### Possible mechanisms of Au/CuS NPs’ antimicrobial activity

**Figure 6:**
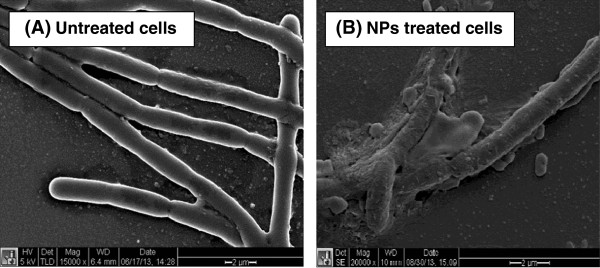
**The SEM images of (A) the control *B. anthracis *** **cells, and (B) cells treated with 4.15 μM Au/CuS NPs for 30 min.**

A number of mechanisms have been proposed for the antimicrobial activity of NPs to bacterial cells. To understand the inactivation of cells treated with Au/CuS NPs, SEM, EDS, and DNA efflux experiments were performed. Figure 6 shows the SEM images of (A) the control *B. anthracis* cells, and (B) cells treated with 4.15 μM Au/CuS NPs for 30 min. The control cells showed an intact membrane while the cells treated with NPs had disordered and damaged membranes. In EDS analysis (Additional file 1: Figure S2), in addition to the elements in the EDS spectrum of the control cells, the Au/CuS treated samples had peaks for sulphur and copper, which indicated the presence of the nanoparticles on the surfaces of treated cells.

The SEM and EDS results suggested that the NPs damage the cell membranes and eventually cause cell death, which is similar to the mechanism of silver nanoparticles reported by Li et al. [27] who investigated the interactions of silver NPs with *Escherichia coli* cells. In their report, they indicated that silver NPs damaged the cell membrane structure and depressed the activity of some membrane enzymes leading to the death of the cell. Au/CuS NPs seemed to produce the same effect in their interaction with *B. anthracis* cells. The literature suggested that electrostatic interactions between negatively charged bacterial cells and positively charged NPs are necessary for the activity of NPs as antibacterial agents [3,30]. The bacterial cell wall is composed mainly of a thick peptidoglycan layer linked to teichoic acid that gives the cell overall negative charges. The negative charges facilitate interactions between the cell and the positively charged NPs, and such interactions possibly cause the loss of membrane integrity. The damaged membrane likely leads to the entry of substances from the environment which results in an osmotic imbalance, at the same time, the leakage of cytoplasmic content, and the consequent rupture of cells.

**Figure 7:**
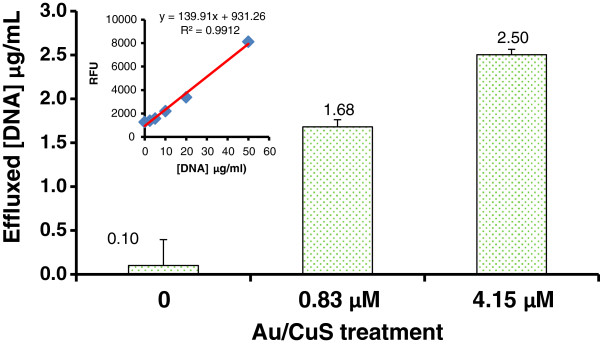
**The amount of DNA effluxed from *B. anthracis *cells treated by 0.83 μM and 4.15 μM Au/CuS NPs for 30 min.** Cell concentration: 1.2×10^7^ cells/mL.

Efflux of cytoplasmic DNA from the treated cells into solutions was examined to determine whether Au/CuS NPs treatment causes bacterial cell membrane damage and the leakage of DNA from treated cells. Figure 7 shows the amount of DNA in solutions after the cells (1.2 × 10^7^ cells/mL) were treated with 0.83 and 4.15 μM Au/CuS NPs for 30 min using the DAPI staining method and quantification with Salmon DNA. The results showed that the cells treated with 0.83 μM Au/CuS NPs leaked 1.68+/−0.08 μg/mL DNA into the buffer and the cells treated with 4.15 μM NPs leaked 2.50+/−0.06 μg/mL DNA into the buffer, suggesting that the bacterial cell membrane was damaged by NPs treatment and that the extent of damage was dependent on NPs concentration.

These observations suggested that the efflux of cytosolic contents from the cells through the damaged membrane was at least a part of the NPs’ antimicrobial mechanism to bacterial cells. Damaging cell membrane is a part of antimicrobial mechanisms for many different NPs [27,29,31]. Other possible mechanisms involved in nanomaterials antimicrobial activity include suppression of energy metabolism [32], inhibition of enzyme activity and induced oxidative stress [29], increased membrane permeability [33-35], and physical piercing [36]. Although these mechanisms might not be all applicable to how Au/CuS NPs damage bacterial cells, it is possible that some of these mechanisms may be involved, as our results confirmed that Au/CuS NPs did inactivate *B. anthracis* cells and inhibit spore outgrowth.

Considering the core/shell structure of the Au/CuS nanoparticles, the shell CuS is the component that most likely interacts with the bacterial cells. With this respect, its mechanisms may be similar to CuS NPs or other Cu compound NPs, but little is known about the antimicrobial activity of pure CuS nanoparticles based on published literature. However, another member in the family of Cu compound, CuO NPs, has recently been reported to show antimicrobial activity to several types of microorganisms [37-39]. Based on the action of CuO NPs and other metal oxide NPs (such as ZnO), the release of soluble metal ions (such as Cu^2+^, Zn^2+^) from the NPs largely influenced its toxicity [37]. In the case of Au/CuS NPs, the NP as a whole could possibly diffuse across the membrane, meanwhile, it is possible that Cu^2+^ solute from NPs can enter cells through transport and ion/voltage-gated channels [40]. While NPs can interact with oxidative organelles or redox active proteins to induce reactive oxygen species (ROS) in cells, Cu^2+^ produced by the NPs can also induce ROS by various chemical reactions, and ROS can break DNA strand and alter gene expression [37]. Other possible mechanism is that Cu^2+^ can form chelates with biomolecules or dislodge the metal ions in some metalloproteins which may lead to dysfunctional proteins and further cell inactivation [37]. However, a surprisingly interesting finding is that CuO NPs’ showed selective antimicrobial activity to different bacteria, they showed dose-dependent inhibitory effect to *E. coli* cells, but not *Salmonella typhimurum*[41]. In another study, CuO NPs–coated cotton fibers showed significant antimicrobial activity to both Gram negative *E. coli* and Gram positive *Staphylococcus aureus*, whereas its analogous CuS-coated cotton fibers did not show any antibacterial activity to these two bacteria [39]. This seems contradictory to the observation in this study that Au/CuS showed antibacterial activity to *B. anthracis* cells, but there is a possibility that Au/CuS NPs may exhibit its antimicrobial activity in a way similar to CuO NPs—that is, selectively to different species of bacteria (since this study used *B. anthracis*, and the other used *E. coil* and S. *aureus*). Nevertheless, the study of Au/CuS NPs’ antimicrobial activity is in a very early stage, its precise mechanisms are not fully understood. Further studies or tests on different bacterial species and in different conditions, systematic comparison with pure Au NPs, CuS NPs, and other similar metal NPs, are necessary to gather more useful information. In addition, examination of the alterations at gene and protein levels will be useful to understand the action mechanisms and will require more comprehensive high-throughput assays such as microarray assay. Certainly, further studies are needed to fully understand the detailed action mechanisms of the antimicrobial activity of Au/CuS to bacterial cells.

## Conclusions

This paper investigated the antimicrobial activity of Au/CuS nanoparticles against *B. anthracis* spores and cells. It was found that Au/CuS NPs were not effective in inactivating *B. anthracis* spores (10^5^ CFU/mL) by pre-treatment ranging from 30 min even up to 24 h. However, the presence of certain levels of NPs (0.0083 μM and higher) in the growth medium can effectively inhibit spore outgrowth, most likely due to NPs inactivation of the vegetative cells that grew from the spores. With great promise, the Au/CuS NPs exhibited much more effective antimicrobial activity against *B. anthracis* cells, and the activity was NPs concentration and treatment time dependent. SEM imaging provided evidence of cell membrane damage and cell death following treatment with Au/CuS NPs. DNA efflux from NPs treated cells confirmed the destruction of cell membranes by NPs. In addition to its PPT effect, this study demonstrated Au/CuS NPs’ antimicrobial activity.

## Methods

### Au/CuS Nanoparticles

The Au/CuS nanoparticles were synthesized in Dr. Chen’s lab at the University of Taxes at Arlington, using the same protocol as previously reported [10]. Briefly, the nanocomposites were synthesized using a two-step procedure. Au nanoparticles were first grown using the seeded growth method and then coated with CuS nanoshell. Detailed reaction conditions and procedures can be found in ref [10]. With the modified amount of reactants, the theoretical concentration of the resulted Au/CuS NPs used in this study was 83 μM. The Au/CuS nanoparticles were 2–5 nm in diameter. The core/shell structure was confirmed by high resolution transmission electron microscope (HRTEM) imaging of the Au and CuS lattice planes in the core and shell, respectively, and by spectrophotometric observation of characteristic absorption peaks for Au and CuS at 531 and 981 nm, respectively [10].

### Preparation of *B. anthracis* Cells and Spores

*B. anthracis* cells were grown by inoculating 50 mL of nutrient broth (pre-warmed to 37°C) (Becton Dickson and Company, Sparks MD) with 200 μL of *B. anthracis* vaccine Sterne 34 F2 (Colorado Vaccine Company, Denver, CO). The culture was incubated at 37°C with 250 rpm agitation in an Excella E25 shaker incubator (New Brunswick Scientific, Edison, NJ) for 18–20 h. The cells were then centrifuged at 4000 × g using a Beckman Coulter Avanti J26XP centrifuge (Beckman Coulter, Atlanta, GA) for 5 min. The supernatant was discarded, and the cell pellet was washed three times with sterile deionized (DI) water, nutrient broth, or PBS. The cells were then resuspended in the respective buffers, and diluted to desired concentrations for further experimental use [20].

Spores were grown by inoculating 100 μL of *B. anthracis* vaccine Sterne 34 F2 into 25 mL of Difco sporulation medium (DSM) prepared according to the method described by Nicholson and Setlow [42]. The culture was incubated in an Excella E25 shaker incubator at 37°C at 150 rpm until it reached mid-log phase (0.45 < A600 < 0.6), followed by adding pre-warmed (37 C) fresh DSM to a total volume of 250 mL, and incubated for an additional 48 h or until > 95% of the culture was free spores. These spores were purified by heating at 70°C for 30 min to kill any remaining vegetative cells and centrifuged at 10,000 × g at 4°C for 10 min. The resulting pellet was washed with cold (4°C) DI-H_2_O eight times to remove cell debris and resuspended in cold DI-H_2_O. The purified spores were stored at 4°C for further experimental use and washed twice with cold water prior to use in each experiment.

### Au/CuS nanoparticle treatment to *B. anthracis* cells and spores

To treat the *B. anthracis* cells or spores with Au/CuS NPs, 900 μL of *B. anthracis* cells or spores (OD_600_nm = 0.9-1, containing 10^5^ spores/mL or 10^7^ cells/mL) were placed in 1.7 mL Eppendorf tubes and added with 1, 10 or 50 μL of Au/CuS NPs and respective buffers to reach a total volume of 1 mL in each tube yielding final Au/CuS NPs concentrations of 0.083, 0.83, and 4.15 μM. The cells or spores in their respective buffers without NPs were used as controls. The samples were rotated at 15 rpm on a Dynal Biotech Rotator (Lake Success, NY) for 0.5, 1, 3, 6, 12 and 24 h at room temperature. After treatment, the growth of treated spores was assessed by the optical density (OD) measurement at 600 nm, and the viable spore or cell number was determined by the traditional plating method.

### OD measurement to assess the growth of NPs treated spores/cells

The NP treated cell or spore samples (1 mL) were transferred to 9 mL of fresh nutrient broth. The samples were then incubated at 37°C and 250 rpm in an Excella E25 shaker incubator. OD_600_ nm was read every hour up to 10 h incubation time using a Spectra Max Plus 384 spectrophotometer (Molecular Devices, Sunnyvale CA). The growth curve for each sample was generated by plotting the OD_600_ nm value against the growth time. To evaluate the growth of the treated samples, their growth curves were compared to that of the controls by comparing the time points at which the exponential phase started.

### The plating method to determine the viable spore/cell number

For the surface plating, the NPs treated samples and control samples were serially diluted by 1:10 in their respective buffers. 100 μL aliquots of appropriate dilution of each sample were plated on Luria Bertani (LB) (Fisher Scientific, Pittsburgh, PA) agar plates. The plates were incubated overnight at 37°C in a Fisher Scientific Isotemp Incubator (Dubuque, Iowa). The number of colonies was counted and the viable spore or cell numbers in the original samples were calculated as colony-forming unit per milliliter (CFU/mL).

### Fluorescence, scanning electron microscopy (SEM) imaging and energy-dispersive X-ray spectroscopy (EDS)

The purpose of imaging was to investigate the interactions between *B. anthracis* cells or spores and Au/CuS nanoparticles. For fluorescence microscope imaging, the cells were treated with 0.83 μM Au/CuS for 10, 20 and 30 min, and stained with a Live/dead BacLight bacteria staining kit (Invitrogen, New York) for 10 min. The staining was done according to the manufacturer’s instructions. Briefly, a 1:1 mixture of the live (SYTO 9) and dead (propidium iodide, PI) stains was prepared, and 3 μL of the mixed dyes were added to 1 mL of each of the samples (*B. anthracis* cells and cells treated with nanoparticles) and incubated at 37°C for 10 min. Then, 5 μL of each stained sample was placed on a microscope slide and covered with the cover slip. The fluorescence images were obtained using a Nikon Eclipse Ti-FLC-E confocal microscope (Japan).

For the SEM imaging, the spores or cells were first treated with 0.83 μM and 4.15 μM Au/CuS nanoparticles for 30 min. The treated spores/cells were fixed in bacterial fixative (4% formaldehyde and 2% glutaraldehyde in 1× PBS). 5 μL of the fixed samples were placed on a cover slide and air dried. The dried sample was then coated with gold using Denton Vacuum Desk IV (Czech Republic) as previously described [20], and the SEM images were taken using the FEI XL30 microscope (Netherlands) at the Shared Materials and Instrumentations Facility (SMIF) at Duke University.

For EDS analysis, the samples were prepared using the same procedure as SEM samples, but without coating with gold. The elemental composition of the samples was obtained by EDS analysis using FEI XL30 ESEM with XFlash 4010 EDS Detector (Czech Republic).

### Efflux of cytoplasmic DNA

Possible cellular membrane damage caused by NPs treatment was examined by measuring intracellular material in the solution of *B. anthracis* cells exposed to Au/CuS NPs. The concentrations of DNA in solutions from cells incubated with and without Au/CuS was determined using the method described by Kang et al. [28] with slight modifications. The cells were treated with 0.083, 0.83 and 4.15 μM Au/CuS for 30 min. The treated sample was centrifuged at 10,000 × g for 4 min. Aliquots of 99 μL supernatant of each sample were put in the wells of a black 96-well plate (Corning Incorporated, NY). Aliquot of 1 μL of 0.5 mg/mL 4, 6-diamidino-2-phenylindole (DAPI) was added to each well at a final concentration of 5 μg/mL. The plate was then incubated in dark at 4°C for 2 h. DAPI has strong binding affinity for the A-T region of DNA, and the intensity of its fluorescence is proportional to DNA concentration. Fluorescence of DAPI at 470 nm in the wells was measured with excitation at 370 nm using the Spectra Max Plus 384 spectrophotometer (Molecular Devices, Sunnyvale CA). Salmon sperm DNA (Amresco, Solon, OH) was used to generate the calibration curves [28] to quantify the amount of DNA in the supernatant of the NPs treated samples.

## Abbreviations

CFU/mL: Colony-forming unit per milliliter; DAPI: 4, 6-diamidino-2-phenylindole; DI water: Deionized water; EDS: Energy dispersive X-ray spectroscopy; FBI: Federal bureau of investigation; HRTEM: High resolution transmission electron microscope; LB agar: Luria bertani agar; NPs: Nanoparticles; OD: Optical density; PTT: Photothermal therapy; PI: Propidium iodide; ROS: Reactive oxygen species; SEM: Scanning electron microscopy.

## Competing interests

The authors declare that they have no competing interests.

## Authors’ contributions

EA, XD, EM carried out the experiments on bacterial cells and spore tests; CY carried out the synthesis of Au/CuS NPs. WC designed and supervised the NPs synthesis experiements. LY designed and supervised the bacterial cells and spores experiments. EA and LY wrote the manuscript. All authors edited and approved the manuscript.

## Supplementary Material

Additional file 1 **The spectra of EDS analysis of untreated** ***B. anthracis*** **spores and spores treated with 4.15 μM Au/CuS for 30 min; and the spectra of EDS analysis of untreated** ***B. anthracis*** **cells and the cells treated with 4.15 μM Au/CuS for 30 min.** Click here for file
