# Supplementary material for: Investigation of antimicrobial activity of photothermal therapeutic gold/copper sulfide core/shell nanoparticles to bacterial spores and cells
Source: J Biol Eng. 2014 Jun 2;8:11. doi: 10.1186/1754-1611-8-11 (PMC4068869; doi:10.1186/1754-1611-8-11)
Supplement: Additional file 1 — The spectra of EDS analysis of untreated B. anthracis spores and spores treated with 4.15 μM Au/CuS for 30 min; and the spectra of EDS analysis of untreated B. anthracis cells and the cells treated with 4.15 μM Au/CuS for 30 min. [file 1754-1611-8-11-S1.docx]

**Additional file 1**

**S1:** The spectra of EDS analysis of untreated *B. anthracis* spores and spores treated with 4.15 µM Au/CuS for 30 min.

**S1**

Untreated Spores

Spores treated with 4.15 µM Au/CuS NPs for 30 min

**S2.** The spectra of EDS analysis of untreated *B. anthracis* cells and the cells treated with 4.15 µM Au/CuS for 30 min.


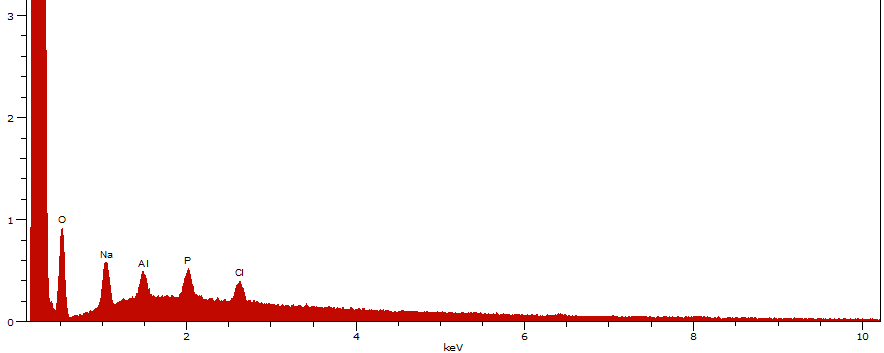


Untreated cells

**
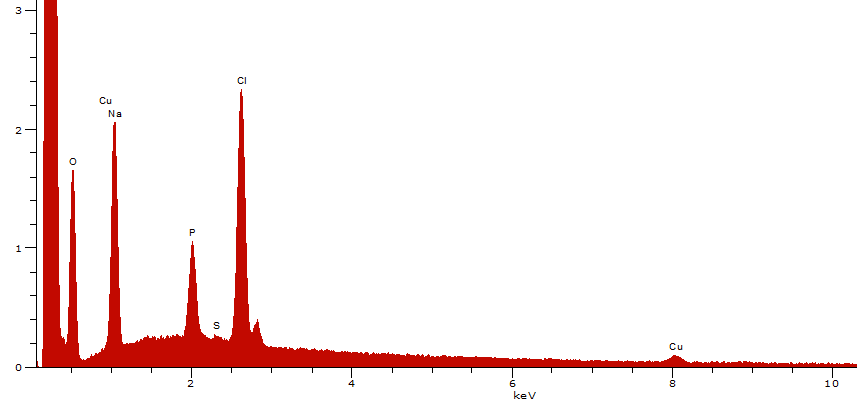
**

Cells treated 4.15 µM Au/CuS NPs for 30 min
